# Prophylaxis of hereditary breast cancer

**DOI:** 10.18632/aging.101342

**Published:** 2017-12-08

**Authors:** Jolien S. de Groot, Paul J. van Diest, Patrick W.B. Derksen

**Affiliations:** Department of Pathology, University Medical Center Utrecht, Utrecht, the Netherlands

**Keywords:** hereditary breast cancer, intraductal intervention, prophylaxis, conditional mouse models

Breast cancer risk increases dramatically in women carrying mutations in a breast cancer susceptibility gene, most frequently *BRCA1*, *BRCA2* or *CHEK2*. Depending on the type of mutation and the contribution of genetic modifiers this risk can increase to more than 80%. Currently, the only effective preventive options for BRCA mutation carriers are highly invasive prophylactic interventions such as bilateral mastectomy and salpingo-oophorectomy. These mutilating procedures have a dramatic psychological and social impact, effect on fertility, and are not always completely effective in preventing hereditary cancer.

**Figure 1:**
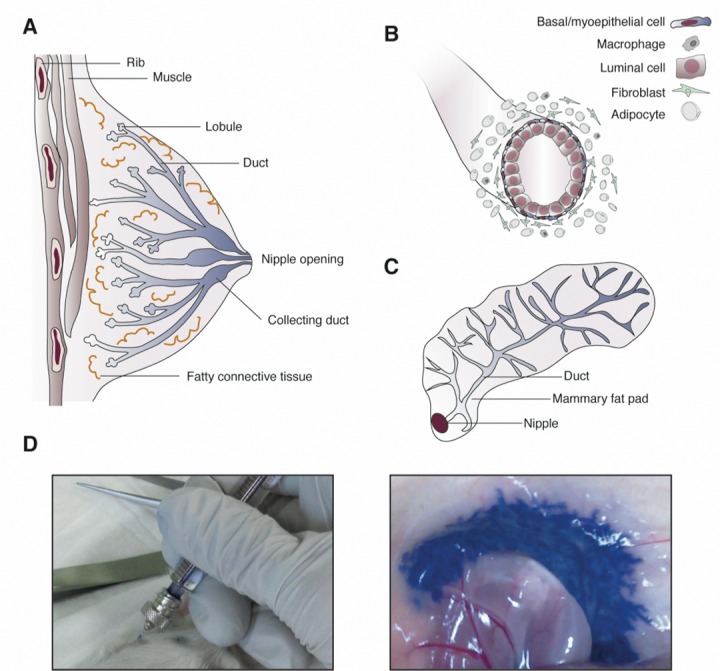
Intraductal injection (**A**) The human breast consists of multiple ductolobular stems that transport the milk produced during lactation in terminal ductolobular units through the breast ducts that surface at the nipple. (**B**) Mammary ducts are bilayered epithelial structures. The inner luminal cells are responsible for milk production and the outer myoepithelial/basal cells contract upon oxytocin release to excrete the milk. (**C**) The murine mammary gland is composed of a branching ductal network localized in the mammary fat pad. The mouse mammary gland has one efferent duct. (**D**) In mice, the nipple can be cannulated with a short, blunt-ended needle (left picture) for ID injection with ablative reagents (indicated in blue; right picture).

An alternative and attractive way to prevent breast cancer development in mutation carriers is a prophylactic treatment of the predisposed mammary epithelial tissue using ablative agents directly via the intraductal (ID) route (Figure 1). Preclinical studies in which ID-administered chemotherapeutics were tested (*i.e*. paclitaxel, pegylated liposomal doxorubicin (PLD), 5-fluorouracil, carboplatin, methotrexate) have shown promising results [1,2]. ID chemotherapy was also explored in phase I trials in women with ductal carcinoma *in situ* and invasive breast cancer prior to mastectomy, where ID administration of PLD and cisplatin was well tolerated with mild adverse events. Moreover, pathological changes could be found in the treated ducts [2,3]. The use of ID administration as a preventive measure is especially appealing in high-risk *BRCA* and *CHEK2* mutation carriers. Since mammary cells deficient in these genes cannot correct stalled DNA replication forks via homologous repair, they are sensitive to platinum-based chemotherapeutics that arrest replication and more sensitive to poly (ADP)-ribose polymerase 1 (PARP1) inhibition. Therefore, we explored the efficacy and safety of ID cisplatin treatment in combination with PARP-inhibition as an alternative prophylactic therapy in the prevention of BRCA1-associated breast cancer [4].

As a first step, we tested if ID cisplatin affected mammary gland reconstitution. Microscopic and Lineage-based fluorescence activated cell sorter analyses showed that cisplatin significantly lowered ductal outgrowth and led to an overall reduction of all mammary epithelial cells. Moreover, ID cisplatin reduced mammary homeostasis and lobulo-alveolar development. Next, we used a conditional mouse model that mimics human *BRCA1*-associated breast cancer (*WAPCre;Brca1F/F;Trp53F/F* mice [5]) to test the preventive effect of ID cisplatin treatment with or without systemic olaparib (PARP-inhibitor). In this set-up we observed that prophylactic ID treatment using cisplatin decreased the onset of breast cancer. Dual therapy using ID cisplatin and olaparib led to a longer tumor-free latency compared to olaparib monotherapy, but not compared to cisplatin monotherapy. Interestingly, tumor-free latency was not only increased in the locally treated, but also in distant untreated mammary glands indicating systemic exposure.

Indeed, platinum was already detected in plasma after 5 minutes and the area under the curve (AUC) values after 96 hours were comparable to that reported after intra-tumoral injection of a similar dose of cisplatin [6]. Moreover, ID injection using high dose cisplatin resulted in an AUC of locally treated glands that was 25% of the treated glands. To serve as an alternative prophylaxis in young BRCA mutation carriers, long-term carcinogenicity of ID cisplatin must be minimal. To test this, we ID injected wild type mice with cisplatin, after which the mice were followed longitudinally until death or disease. Treated mice developed more mammary carcinomas than control mice after long latency periods (679 days), and showed an increased tumor incidence in distant untreated glands. Of note, we observed that the incidence of primary lung adenocarcinomas of the lung was also increased in treated mice. In conclusion, although very promising as a prophylactic treatment strategy, the two main issues that interfered with successful ID cisplatin prophylaxis in mice were incomplete tumor prevention and the systemic exposure leading to long-term side effects.

ID injection of the mammary ductal system is a delicate procedure. First, proximal branching of the multiple efferent ducts may hinder fully penetrant treatment. Also, treatment will be dependent on hormonal fluctuations during menstrual cycle stages, and because the latter affects epithelial proliferation and therefore sensitivity to cisplatin drugs, this might influence cytotoxic susceptibility. Finally, intrinsic resistance to cisplatin or olaparib could explain therapy resistance.

A major shortcoming of ID cisplatin is the observed systemic exposure, which has also been observed in preclinical and clinical trials using other chemotherapy regimens [2,3]. Further, platinum can be released from regenerating tissues leading to circulating platinum even after two decades post treatment [7]. Hence, when developing a safe intervention to prevent hereditary breast cancer in young and healthy mutation carriers, the ablative agent should stay strictly localized to the ductal system during treatment and be rendered inactive afterwards. If these criteria are met, we propose that cancers in mutation carriers could be prevented by intraductal epithelial ablation. It is still a matter of debate whether the best timing of ID-treating mutation carriers should be performed just prior to the first menarche or following the completion of breast feeding for the final child. Regardless of the exact timing, alternative treatments using immobilized ablative agents based on thermal, optical or radionuclides should be explored to assess their efficacy in preventing hereditary breast cancer. For this, we believe that a combination of proof-of-principle tests in human samples, combined with conditional mouse models of breast cancer and long-term follow-up in a non-malignant setting would be the preferred and effective set-up.
